# The role of theory and modeling in medical research

**DOI:** 10.3389/fphys.2013.00377

**Published:** 2013-12-19

**Authors:** Olaf Wolkenhauer

**Affiliations:** ^1^Department of Systems Biology and Bioinformatics, Institute of Computer Science, University of RostockRostock, Germany; ^2^Wallenberg Research Centre, Stellenbosch Institute for Advanced Study, Stellenbosch UniversityStellenbosch, South Africa

**Keywords:** systems biology, mathematical modeling, theory, systems medicine, medicine

Computers can be used to conduct experiments that would otherwise be difficult in the lab, if not impossible. An example is the study of the role specific atoms play at different stages in a chemical reaction. An intuitive approach would be to scale up processes at the atomic level to create simulations of large molecules and their reactions. Despite advances in computer power this is not possible. For each level of organization there are tools and methodologies available and the clever combination of these two worlds brought the breakthrough to computationally efficient multi-scale simulations that was awarded the 2013 “Nobel Prize in Chemistry.” (www.nobelprize.org) Now, take the current developments in medical research, where researchers are trying to explain complex physiological phenomena at the tissue and organ level with insights gained from experiments at the molecular and cellular level. Research efforts, like the Virtual Physiological Human or the Human Brain Project, are under way to bridge the molecular with the physiological world and one wonders whether one can learn something from the Nobel Prize. What are the best strategies for the development of multiscale models for complex physiological systems?

Mathematical modeling and computer simulations of complex biological systems has a long history and in combination with wet-lab experiments, such systems biology approaches have been quite successful in complementing bioinformatics approaches in the study of cellular systems. Under the emerging notion of “Systems Medicine” the goals for multiscale modeling are set high—aiming for a linkage between the molecular, cellular, tissue, organ, and whole organism levels (Wolkenhauer et al., 2013). As in chemistry, bottom-up approaches are unlikely to succeed. Instead, an integration of methodologies at different levels seems more promising. This, however, requires not only computer simulations but also the development and application of mathematical concepts for the theoretical discovery of mechanisms and organizing principles that can subsequently be explored in experiments.

The 2013 Nobel Prize in Physics demonstrates the role theory can play. Mathematics provides not only foundation for the construction and simulation of models but it can also be used to formulate law-like organizing principles. Here mathematical theory is providing a way of thinking, helping us to formulate hypotheses about nature, that may later be confirmed through experiments and with the help of computer simulations. While simulations can be systematically planned and executed, such theoretical developments rely more heavily on original insights. There are many mechanisms that can be found but only few law-like organizing principles to be discovered (Wolkenhauer et al., 2011; Wolkenhauer and Green, 2013). While the 2013 Nobel Prize in Chemistry emphasizes the role multiscale modeling and simulation could play in biomedicine, the prize in physics demonstrates the way theory can guide experiments, and conversely how experiments inform theory. The theory that was awarded the 2013 Nobel Prize in Physics was 48 years ahead of the experiments that confirmed it. Complex systems require long term multidisciplinary collaborations and the experiments that confirmed the predicted existence of a Higgs particle are also an impressive sociological achievement, which provides lessons for medical research. Several thousand scientists from around the world participated in the experiments. Systems Medicine is an interdisciplinary approach to understand and treat diseases and its success will heavily rely on our ability to integrate and coordinate large-scale research efforts. While theories of tissue organization can be developed by individuals, their confirmation requires long term coordinated efforts. There is no doubt that biological systems can match the complexity of physical systems. Surely any disease generates questions as challenging as the confirmation of the Higgs particle.

It will thus be some time before we see a Nobel Prize in Medicine awarded for the development of multiscale simulations of complex cellular systems, or the development and confirmation of a theory of tissue (mal)function but the work to deserve such a prize should be underway now.
